# Dysregulation of Treg/Th17 Balance and Intracellular Expression of IL-21 and IL-22 in the Pathogenesis of Gestational Hypertension

**DOI:** 10.3390/jcm14207288

**Published:** 2025-10-15

**Authors:** Maciej Kwiatek, Wojciech Kwaśniewski, Tomasz Gęca, Ewelina Grywalska, Mansur Rahnama-Hezavah, Sebastian Mertowski, Tomasz Urbanowicz, Magdalena Ewa Kowalkowska, Maciej Krasiński, Anna Kwaśniewska, Maciej Brązert

**Affiliations:** 1 Department of Obstetrics and Pathology of Pregnancy, Medical University of Lublin, 16 Staszica St, 20-081 Lublin, Poland; maciej.kwiatek@umlub.pl (M.K.); tomasz.geca@umlub.edu.pl (T.G.); anna.kwasniewska@umlub.pl (A.K.); 2 Department of Oncological Gynecology and Gynecology, Medical University of Lublin, 20-081 Lublin, Poland; wojciech.kwasniewski@umlub.pl; 3 Department of Experimental Immunology, Medical University of Lublin, 4a Chodźki Street, 20-093 Lublin, Poland; ewelina.grywalska@umlub.pl; 4 Department of Dental Surgery, Medical University of Lublin, 6 Chodźki Street, 20-093 Lublin, Poland; mansur.rahnama@umlub.pl; 5 Cardiac Surgery and Transplant Medicine Department, Poznan University of Medical Sciences, 61-848 Poznan, Poland; turbanowicz@ump.edu.pl; 6 Clinical Department of Oncologycal Genecology, Oncology Centre in Bydgoszcz, 85-796 Bydgoszcz, Poland; mg.kowalkowska@gmail.com; 7 Thoracic Research Centre, Collegium Medicum UMK, 85-067 Bydgoszcz, Poland; 8 Department of Obstetrics and Gynaecology, St Mary’s Hospital, Imperial College Healthcare Trust, London W2 1NY, UK; mkrasinski975@gmail.com; 9 Department of Diagnostics and Treatment of Infertility, Poznan University of Medical Sciences, 60-535 Poznan, Poland; maciejbrazert@ump.edu.pl

**Keywords:** Th17 cells, regulatory T cells, IL-21, IL-22, pregnancy-induced hypertension, preeclampsia, flow cytometry, cytokines, immune regulation, maternal immunity

## Abstract

**Background/Objectives:** Pregnancy-induced hypertension (PIH), including preeclampsia (PE), remains a significant cause of maternal and fetal morbidity. Immune imbalance involving T helper (Th17) and regulatory T (Treg) cells is increasingly recognized as contributing to the pathogenesis of PIH. This study aimed to assess the proportions of Th17 and Treg cells and intracellular cytokine expression (IL-17A, IL-17F, IL-21, and IL-22) in the peripheral blood of hypertensive versus normotensive pregnant women. **Methods:** A total of 108 pregnant women were included: 60 with hypertensive disorders and 48 normotensive controls. Peripheral blood mononuclear cells were analyzed using multiparametric flow cytometry to quantify CD4+CD25+FoxP3+ Treg and CD4+IL-17A+ Th17 cells, along with intracellular IL-17F, IL-21, and IL-22 co-expression. Correlations with clinical and obstetric parameters were evaluated. **Results:** Hypertensive patients showed significantly increased proportions of activated Th17 cells (CD4+IL-17A+) and Th17 subpopulations co-expressing IL-17F and IL-22, as well as IL-21 and IL-22 (*p* < 0.0001). Although Treg cell percentages were lower in the hypertensive group, the difference was not statistically significant. A pronounced Th17/Treg imbalance was observed. Positive correlations were found between Th17 subpopulations and gestational age, birth weight, and length, as well as maternal age. **Conclusions:** The immune profile in hypertensive pregnancies was characterized by a shift toward Th17-mediated proinflammatory responses, supporting the role of immune dysregulation in PIH. The increased frequency of Th17 cells co-expressing IL-21 and IL-22 may serve as a potential biomarker of disease severity and warrants further exploration.

## 1. Introduction

Maintaining a healthy pregnancy relies on a dynamic yet balanced interaction between maternal immunity and the developing fetus [1]. One key element of this balance is the relationship between proinflammatory Th17 lymphocytes and immunosuppressive Treg lymphocytes. Disruption of this balance exacerbates inflammation, promotes endothelial dysfunction, and compromises placental perfusion—phenomena relevant to pregnancy-induced hypertension (PIH) and preeclampsia [2,3,4,5,6].

Th17 and Treg cells originate from the same pool of naive CD4^+^ lymphocytes, and their differentiation direction is determined by local cytokines. TGF-β promotes FoxP3 expression and Treg development, while the presence of IL-6 or IL-1β activates RORc and shifts differentiation toward Th17 [7,8,9,10]. IL-21 further enhances this shift by inhibiting FoxP3 and promoting Th17 development independently of IL-6 (confirmed in vivo) [11]. Tregs, in turn, limit Th17 activation by inhibiting IL-1β/IL-6/IL-23, inducing IL-27 in dendritic cells, and secreting IL-10 and IL-35 [12,13,14,15]. TGF-β can also downregulate IL-23R expression on Th17 cells, promoting a regulatory phenotype [13,16]. Th17 cells maintain plasticity—in the presence of IL-12 or IL-4, they adopt Th1-like (IL-17^+^/IFN-γ^+^) or Th2-like (IL-17^+^/IL-4^+^) characteristics, respectively. Co-expression of chemokine receptors (CCR4/CCR5/CCR6/CXCR3/CXCR6) indicates tight connections and migration between Th1/Th17/Treg subpopulations [8,17]. Activated Th17 secrete IL-17A and IL-17F, as well as IL-6, IL-21, IL-22, and TNF-α [18,19,20]. An excess of these cytokines is associated with the activation of angiotensin type 1 receptors, oxidative stress, and vasoconstriction, which clinically translates into increased blood pressure in pregnancy [21]. IL-21 and IL-22 (along with IL-17) are of particular interest. IL-21 expression and the availability of its receptor increase under inflammatory and autoimmune conditions [22], and IL-21 itself has pleiotropic effects [23,24]. IL-22 can act in two ways—proinflammatory or regenerative—primarily affecting epithelial and stromal cells, supporting tissue repair and barrier integrity [25,26]; At the same time, it may enhance IFN-γ in Th1/Tc1 and limit IL-10 in Treg/Th2, enhancing the cytotoxic response [27]. Reports of higher IL-22 concentrations in preeclampsia than in normal pregnancies further indicate the involvement of this axis in pathogenesis [28]. Despite growing knowledge of the Th17/Treg system, the mechanisms underlying its imbalance in PIH are not fully understood [20].

Therefore, the aim of our study was to assess the population of Treg (CD4^+^CD25^+^FoxP3^+^) and Th17 lymphocytes, as well as the intracellular expression of IL-17A, IL-17F, IL-21, and IL-22 in women with PIH (including preeclampsia). By focusing on cytokines potentially affecting endothelium and vessels (IL-17A/F) and immunoregulation (IL-21/IL-22), we aimed to clarify their role in the immunopathogenesis of PIH and assess their usefulness as immunological risk markers.

## 2. Materials and Methods

### 2.1. Study Design and Group Characteristics

We conducted an observational, prospective case–control study including pregnant women with pregnancy-induced hypertension (cases) and normotensive pregnant women (controls). The project received a positive opinion from the Bioethics Committee of the Medical University of Lublin (Resolution No. KE-0254/19/2012 of 26 January 2012), and its implementation was conducted in accordance with the principles outlined in the Declaration of Helsinki. All study participants signed an informed consent form.

The study included patients hospitalized at the Clinic of Obstetrics and Pathology of Pregnancy, Medical University of Lublin, between January 2013 and December 2023. The study group consisted of 60 pregnant women diagnosed with hypertension after the 20th week of pregnancy according to the criteria of the Polish Society of Gynecologists and Obstetricians [29].

Inclusion criteria also included the absence of antihypertensive treatment before pregnancy. The control group consisted of 48 women with physiological, full-term pregnancies (≥37 weeks) and without obstetric complications, who had blood pressure values within normal limits throughout pregnancy. Verification of obstetric complications was based on the complete medical records of recruited patients, including pregnancy records, hospitalization history, and laboratory and imaging test results. Additionally, a normal pregnancy was confirmed through data on delivery at ≥37 weeks, normal blood pressure throughout pregnancy, and neonatal outcomes, such as birth weight appropriate for gestational age and normal Apgar scores.

Exclusion criteria included:

- Chronic hypertension,
- Kidney and liver disease,
- Autoimmune diseases,
- Active or previous cancer,
- Multiple pregnancies,
- Preterm births,
- Evidence of systemic infection at the time of blood sampling.

Basic demographic and clinical data were analyzed based on medical records: patient age, height, weight, BMI, obstetric and family history, gestational age at delivery, systolic blood pressure (SBP) and diastolic blood pressure (DBP), as well as neonatal parameters (birth weight, length, Apgar score at 1 and 5 min of life).

### 2.2. Biological Sample Collection

Venous blood (approximately 5 mL) was collected from each patient into sterile lithium heparin tubes (BD Vacutainer^®^ Becton Dickinson, Franklin Lakes, NJ, USA ) under aseptic conditions. The collection was performed in the morning, after an empty stomach and rest, to minimize the impact of stress factors on the cytokine profile. Samples were processed within a maximum of 2 h of collection. The procedure involved the separation of peripheral blood mononuclear cells (PBMCs) by gradient centrifugation and subsequent cell labeling for flow cytometric analysis. The procedure involved the separation of peripheral blood mononuclear cells (PBMCs) using Gradisol L (Aqua-Med, Łódź, Poland) according to the manufacturer’s procedure and descriptions available in the scientific literature. The blood sample was diluted 1:1 with calcium- and magnesium-free PBS buffer and then gently transferred onto a separation medium layer. The sample was centrifuged at 700× *g* for 20 min at room temperature, and the resulting PBMC-rich layer was then removed with a sterile pipette and washed twice in PBS (10 min, 400× *g*, 4 °C) to remove residual gradient solution and cellular components.

### 2.3. Immunophenotyping of Regulatory T Cells (Treg)

Three-color flow cytometry was used to assess the Treg subpopulation from PBMC using a FACSCanto™ II cytometer (Becton Dickinson, Franklin Lakes, NJ, USA) and CellQuest™ Pro software (version 6.0; Becton Dickinson, Franklin Lakes, NJ, USA) analytical software. The procedure involved surface and intracellular staining using a commercial panel of monoclonal antibodies:

- Alexa Fluor^®^ 647 Mouse anti-Human FoxP3 (BD Biosciences, San Diego, CA, USA),
- V450 Mouse anti-Human CD4 (BD Biosciences, San Diego, CA, USA),
- PE-Cy™7 Mouse anti-Human CD25 (BD Biosciences, San Diego, CA, USA).

**Figure 1:**
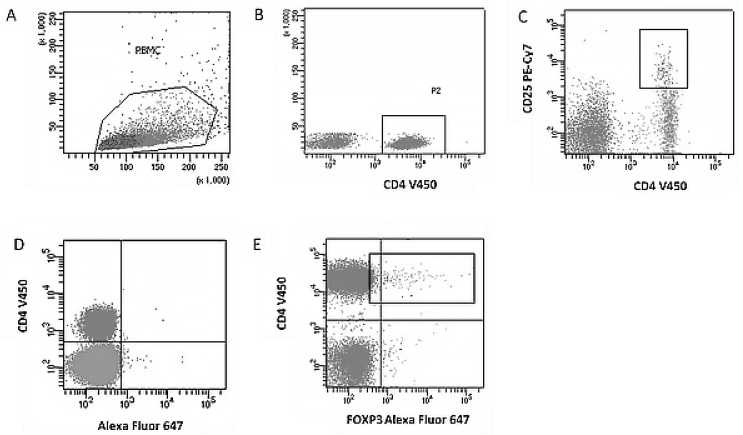
Example of flow cytometric analysis of a regulatory T cell subpopulation (CD4+CD25+FoxP3+). (**A**). Dot plot presenting light scatter parameters (FSC/SSC) on a linear scale, enabling the isolation of the PBMC (peripheral blood mononuclear cells) region and the selection of lymphocyte populations. (**B**). FSC/SSC plot with additional analysis in the SSC system (linear scale) versus CD4 marker expression (V450 channel, logarithmic scale), enabling the creation of gate R2 containing CD4+ cells. (**C**). Dot plot in a logarithmic system (CD4 V450 vs. CD25 PE-Cy7) presenting the coexpression of CD4 and CD25, enabling the isolation of the CD4+CD25+ T cell population. (**D**). Control plot using Mouse IgG1 isotype antibody (Alexa Fluor 647) versus CD4 (V450) expression to define the cut-off point for positive expression of the FoxP3 intracellular antigen. (**E**). Dot plot (CD4 V450 vs. FoxP3 Alexa Fluor 647) on a logarithmic scale illustrating the final identification of the regulatory T cell population (CD4+CD25+FoxP3+) with intracellular expression of the FoxP3 transcription factor.

The staining process involved cell permeabilization using the Fix/Perm Buffer Set (BD Biosciences, San Diego, CA, USA) according to the manufacturer’s instructions and an isotype control (IgG1-Alexa Fluor 647) to determine the cutoff point. Treg cells were defined as CD4+CD25+FoxP3+. The CD4+ lymphocyte population was distinguished based on the FSC (forward side channel)/SSC (scatter side channel) region and CD4 expression, and co-expression of CD25 and FoxP3 was analyzed (Figure 1).

### 2.4. Analysis of Th17 Subpopulations and Cytokine Expression

To quantitatively analyze cytokine expression in the Th17 lymphocyte subpopulation, the Human Th17 Cytokine Staining Panel (eBioscience, Thermo Fisher Scientific, Waltham, MA, USA) was used, including:

- FITC anti-human IL-17A (eBio64Dec17),
- PE anti-human IL-17F (SHLR17),
- PerCP-eFluor^®^ 710 anti-human IL-22 (22URTI),
- Alexa Fluor^®^ 647 anti-human IL-21 (FFA21),
- eFluor^®^ 450 anti-human CD4 (RPA-T4).

**Figure 2:**
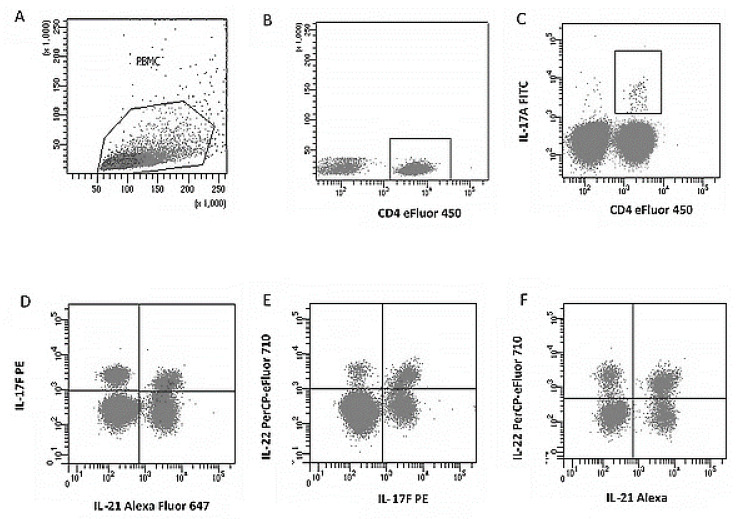
Example of flow cytometric analysis of the Th17 lymphocyte subpopulation with intracellular expression of IL-17F+, IL-21+, IL-17F+, IL-22+, and IL-21+, IL-22+ in the peripheral blood lymphocyte population. (**A**). Dot-plot (FSC vs. SSC) showing the light scattering characteristics of the cells (linear scale) to isolate the peripheral blood mononuclear cell (PBMC) population and to create a region containing lymphocytes. (**B**). Dot-plot of SSC (linear scale) versus CD4 marker expression (eFluor^®^ 450, logarithmic scale) to enable gating of the CD4+ T lymphocyte subpopulation. (**C**). Logarithmic dot-plot showing co-expression of CD4 (eFluor^®^ 450) and IL-17A (FITC), used to identify the Th17 cell population (CD4+IL-17A+). (**D**). Dot plot (IL-17F PE vs. IL-21 Alexa Fluor^®^ 647) on a logarithmic scale, illustrating the Th17 cell subpopulation simultaneously expressing intracellular IL-17F+ and IL-21+. (**E**). Logarithmic dot-plot (IL-17F PE vs. IL-22 PerCP-eFluor^®^ 710), used to identify Th17 cells co-producing IL-17F+ and IL-22+. (**F**). Logarithmic dot plot (IL-21 Alexa Fluor^®^ 647 vs. IL-22 PerCP-eFluor^®^ 710) showing the Th17 subpopulation with simultaneous expression of IL-21+ and IL-22+.

PBMC obtained by the concentration gradient method described above were suspended in RPMI-1640 (Gibco, Grand Island, NY, USA) with 10% FBS (Gibco, Thermo Fisher Scientific, Waltham, MA, USA), 2 mM L-glutamine (Gibco, Thermo Fisher Scientific, Waltham, MA, USA), 100 U/mL penicillin and 100 µg/mL streptomycin (Gibco, Thermo Fisher Scientific, Waltham, MA, USA), and 10 mM HEPES (Gibco, Thermo Fisher Scientific, Waltham, MA, USA) and incubated at 37 °C, 5% CO_2_, in an atmosphere of 100 mM HEPES. For cytokine induction, cells were plated at 1.0 × 10^6^ cells. The cultures thus established were stimulated with PMA (50 ng/mL; Sigma-Aldrich, St. Louis, MO, USA) and ionomycin (1 µg/mL; Sigma-Aldrich, USA) for 4 h (37 °C/5% CO_2_). To inhibit protein transport, brefeldin A (5 µg/mL; eBioscience™, Thermo Fisher Scientific, Waltham, MA, USA) was added at the start of stimulation. Unstimulated cultures were established in parallel as a negative control. After activation of cells with the PMA/ionomycin stimulator in the presence of a protein transport inhibitor (brefeldin A), permeabilization and intracellular staining were performed. The Th17 cells were defined as CD4+IL-17A+, and the percentage of cells dual-expressing IL-17F+/IL-21+, IL-17F+/IL-22+, and IL-21+/IL-22+ was analyzed. Gates were set based on isotype controls and unequivocally positive populations (Figure 2).

### 2.5. Statistical Analysis

Statistical analyses were performed using MedCalc (v.15.8 PL MedCalc Software Ltd., Ostend, Belgium) and Statistica (v.13 PL, TIBCO Software Inc., Santa Clara, CA, USA) software. The normality of the distribution of continuous variables was verified using the D’Agostino–Pearson test. Due to the non-normal distribution of data, nonparametric tests were used:

- Mann–Whitney U test—for comparisons between groups.
- Spearman’s rank correlation test—for the analysis of relationships between continuous variables. The strength of the correlation between two variables in a given test was described based on the correlation coefficient (rho). Rho values between 0.1 and 0.3 are considered weak. Values between 0.3 and 0.5 are considered moderate, while coefficients above 0.5 suggest a strong correlation.
- Categorical data were presented as absolute numbers and percentages (%).

The study sample size was assessed with the endpoint of Th17 lymphocyte percentage (CD4^+^IL-17A^+^) in peripheral blood as the target. A non-normal distribution was assumed, so the power plan was based on the Mann–Whitney test (independent, two-sided differences). Based on pilot data from our population and literature results, a predicted effect size of AUC = 0.70 (corresponding to a moderate effect in terms of Cliff’s delta ≈ 0.40) was assumed. For α = 0.05 and power of 0.80, the minimum sample size was ≥36 subjects per group. Taking into account potential missingness/exclusions (≈20%), recruitment of at least 45 subjects per group was planned. Ultimately, 60 PIH patients and 48 controls were recruited, which exceeds the required minimum and provides a power of ≥0.80 even with an unequal sample distribution (allocation coefficient k ≈ 1.25). Calculations were performed with G*Power 3.1 (Heinrich Heine Universität, Düsseldorf, Germany).

All statistical tests were two-sided. A level of *p* < 0.05 was considered statistically significant.

## 3. Results

### 3.1. Clinical and Demographic Characteristics of the Patients, Family History, and Blood Pressure Levels

Analysis of demographic data revealed that patients in both groups (study and control) did not differ significantly in age, number of pregnancies, parities, or spontaneous abortions. However, significant differences were noted in gestational age at delivery. In the study group, the mean gestational age was significantly lower than in the control group (38 vs. 40 weeks; range: 24–41 vs. 37–41 weeks; *p* < 0.0001), indicating a higher incidence of preterm birth among patients with pregnancy-induced hypertension.

Both maternal prepregnancy weight and body mass index (BMI) were significantly higher in the study group compared to the control group. The median pre-pregnancy body weight was 69.5 kg vs. 60 kg (*p* = 0.0002), while the median BMI was 24.0 vs. 21.9 kg/m^2^ (*p* = 0.0003). At the time of sample collection, body weight (85 kg vs. 75.5 kg; *p* = 0.0001) and BMI (30.7 vs. 26.8 kg/m^2^; *p* = 0.0001) remained significantly higher in the study group, which may reflect the coexistence of overweight or obesity as a risk factor for the development of gestational hypertension.

Analysis of data regarding family history of hypertension revealed a higher rate of positive history in the study group (31.7%) compared to the control group (14.6%) (Table 1). However, this difference did not reach statistical significance (*p* = 0.0662). Statistically significant differences were observed between the groups in terms of blood pressure values, both before and during pregnancy. Systolic blood pressure (SBP) before pregnancy was significantly higher in the study group (median: 120 mmHg [IQR: 120–130]) compared to the control group (120 mmHg [IQR: 110–120]; *p* = 0.0014). The differences were even more pronounced in diastolic blood pressure (DBP), which was 80 mmHg [IQR: 80–85] before pregnancy, compared to 71.5 mmHg [IQR: 68–80] after pregnancy; *p* < 0.0001.

During pregnancy, SBP and DBP values further increased in the study group. Median SBP was 155 mmHg [IQR: 145–165] vs. 120 mmHg [IQR: 113.5–122.5] in the control group (*p* < 0.0001), while median DBP was 95.5 mmHg [IQR: 90–105] vs. 80 mmHg [IQR: 70–80]; *p* < 0.0001. These results confirm the presence of clinically significant hypertension in the study group. A summary of the results is provided in Table 1, which includes a comparison of family history and systolic and diastolic blood pressure values before and during pregnancy (Table 1).

### 3.2. Immune Profile—Th17 and Treg Lymphocyte Subpopulations

T-helper cell immunophenotype analysis revealed significant differences in the percentage of activated Th17 lymphocytes and their subpopulations between patients with pregnancy-induced hypertension and women with a normal pregnancy (Table 2). A significantly higher percentage of activated Th17 lymphocytes (CD4+IL-17A+ phenotype) was observed in the study group compared to the control group (median: 6.8% vs. 1.8%; *p* < 0.0001), as confirmed by flow cytometric analysis. Furthermore, in the group of patients with gestational hypertension, a significantly increased percentage of Th17 cells co-expressing IL-17F and IL-22 was observed (median: 2.7% vs. 0.7%; *p* < 0.0001), as well as a Th17 subpopulation with intracellular expression of IL-21 and IL-22 (median: 1.7% vs. 0.8%; *p* = 0.0001). At the same time, the percentage of regulatory T cells (CD4+CD25+FoxP3+) was lower in the study group compared to the control group (median: 1.9% vs. 2.4%); however, this difference did not reach statistical significance (*p* = 0.1677). In turn, the Treg/Th17 ratio, expressing the ratio of immunosuppressive to effector cells, was significantly lower in the study group (median: 5.5) than in the control group (median: 19.3; *p* < 0.0001), reflecting a significant shift in the immune balance towards pro-inflammatory responses. Regarding the remaining analyzed immunological parameters in peripheral blood, including the overall percentage of lymphocytes and Th17 lymphocytes co-producing IL-17F and IL-21, no statistically significant differences were observed between the groups (*p* = 0.9501 and *p* = 0.8368, respectively). Detailed data are presented in Table 2.

### 3.3. Correlation Analysis Between Immunological Parameters and Demographic and Clinical Variables

In a correlation analysis conducted in a group of patients with pregnancy-induced hypertension, significant positive correlations were observed between the percentages of selected Th17 lymphocyte subpopulations and demographic and clinical variables (Table 3). The percentage of Th17 lymphocytes with intracellular expression of IL-17F and IL-21 correlated positively with gestational age at the date of biological sample collection. The Spearman’s rank correlation coefficient was rho = 0.263 with a significance value of *p* = 0.0484, indicating a weak but statistically significant correlation. This may suggest that with advancing gestational age, a physiological or pathologically modulated increase in this lymphocyte subpopulation occurs in women with gestational hypertension. Even stronger correlations were observed for Th17 lymphocytes co-producing IL-17F and IL-22. The percentage of these cells showed a positive correlation with both the gestational week on the day of sample collection (rho = 0.325; *p* = 0.0136) and the week of pregnancy termination (rho = 0.333; *p* = 0.0114). Furthermore, these lymphocytes showed a positive correlation with anthropometric parameters of the newborns. Birth weight was significantly correlated with their percentage (rho = 0.328; *p* = 0.0128), and neonatal body length exhibited a moderate positive correlation (rho = 0.404; *p* = 0.0020). These relationships suggest that the expansion of this specific Th17 subpopulation is linked to both gestational age and fetal maturation. They may also play a compensatory role in immune development against the backdrop of the disturbed inflammatory profile in patients with PIH.

In the case of Th17 lymphocytes simultaneously expressing IL-21 and IL-22, a positive correlation was observed with maternal age (rho = 0.335; *p* = 0.0109), suggesting that this type of immune response may be more strongly represented in older pregnant women with gestational hypertension. Regarding other immunological parameters, including the percentage of Tregs (CD4+CD25+FoxP3+), the total number of activated Th17 (CD4+IL-17A+), and the Treg/Th17 ratio, no significant correlations were observed with the analyzed clinical and demographic variables. Similarly, the number of pregnancies, deliveries, and miscarriages did not demonstrate significant associations with any of the analyzed immunological parameters.

## 4. Discussion

For years, pregnancy was described as a state dominated by Th2 responses and anti-inflammatory factors. Today, we know that it is rather a dynamic balance in which pro- and anti-inflammatory responses complement each other [30]. In the early first trimester (implantation, placental development), a physiological inflammatory phase is necessary. This is evident in the higher percentage of Th17 cells in the decidua than in the endometrium during the secretory phase [31,32,33,34]. In the following weeks, the profile shifts toward a more anti-inflammatory one: in the second trimester, the number of Tregs increases, and then gradually declines; just before delivery, Th17 and inflammatory cytokines increase again, likely facilitating the onset of labor [31,35,36]. Treg levels 6–8 weeks after delivery are only slightly higher than before pregnancy [35]. Disruption of the Treg/Th17 axis plays a role in the pathogenesis of pregnancy-induced hypertension (PIH) [20]. In normal pregnancy, both subpopulations function within a broader cellular network, maintaining homeostasis, whereas in PIH, both the Treg/Th17 balance and the CD4^+^ cytokine profile are disturbed [5,8,20]. The Th17/Treg ratio tends to increase throughout pregnancy and peaks in the postpartum period; some authors attribute the decline in circulating Tregs at the end of pregnancy to their migration to the decidua [8,20]. The persistent shift toward Th17 may therefore reflect a physiological pro-inflammatory state promoting labor and partially explain the higher incidence of preeclampsia in the second half of pregnancy [20,37]. Accordingly, women with preeclampsia (PE) have a higher expression of Th1/Th17 factors (T-bet, RORc) and a lower expression of Treg/Th2 factors (FoxP3, GATA-3) than in normal pregnancies [38,39,40]. During pregnancy, both the decrease in Treg numbers and deterioration of their function may weaken tolerance to paternal antigens and disrupt the formation of an immunologically privileged environment in the decidua [41,42]. In turn, excessive Th17 activation and increased levels of proinflammatory cytokines (IL-17, IL-21, IL-22) trigger intensive recruitment of effector cells that secrete further inflammatory mediators (including IL-1, IL-6, IL-8, IFN-γ, TNF-α) [37,43]. This picture supports the hypothesis that generalized inflammation in preeclampsia results from an imbalance between the Th2/Treg and Th1/Th17 axes [35,40]. Most studies show that in normal pregnancy, Th17 cells constitute a small percentage with increased Treg numbers, whereas in preeclampsia, the Treg/Th17 balance shifts towards a Th17 predominance [35,40].

In our material, the percentage of all peripheral blood lymphocytes in patients with gestational hypertension did not differ significantly from the values in a group of healthy pregnant women—similar to Fu et al., who observed no differences in the main subpopulations (T, NK, monocytes, granulocytes), except a lower number of B lymphocytes in preeclampsia [44]. At the same time, in hypertension disorders in pregnant women, lower Treg activity, a shift towards Th1, and a greater presence of Th17 have been described [45].

The shift towards Th17 responses in PIH that we observed is consistent with the literature [20,35,46]. Unlike some studies, however, we did not observe significant differences in the total percentage of Tregs between PIH and controls. The increased inflammation accompanying PIH may create an autocrine vicious cycle that drives further Th17 production. High concentrations of TGF-β and elevated IL-6 in the microenvironment promote the differentiation of naive T lymphocytes into Th17 [10]. Our results (higher percentage of CD4^+^IL-17^+^) are consistent with reports of increased CD4^+^IL-17A^+^ cells in blood and increased RORc mRNA expression in the decidua and PBMCs in women with gestational hypertension, including preeclampsia [28,35,40,43,46,47,48]. In vitro data also indicate that human Th17 differentiation/proliferation requires IL-1 and IL-6 and/or IL-21 and is inhibited by high concentrations of TGF-β and IL-6 or IL-21 [49]. Concurrently, monocytes from women with preeclampsia produce more IL-1 and IL-6 than monocytes from healthy pregnant women [50]. Finally, an imbalance in angiogenic factors may further promote PIH and fetal growth disorders [51,52]. These factors are crucial for trophoblast implantation and villi transformation into the placenta [53]. Clinically, elevated sEng levels are observed in both early and late preeclampsia; high endoglin blocks the action of TGF-β, facilitating CD4^+^ differentiation toward Th17 [50,54]. Moreover, higher placental expression and circulating levels of sEng and sFlt-1 positively correlate with the severity of preeclampsia [55].

In women with pregnancy-induced hypertension, a higher percentage of Th17 cells (CD4^+^ IL-17^+^) suggests the involvement of this subpopulation in the generalized inflammatory response in the circulation. IL-17 secreted by Th17 triggers a cascade of subsequent mediators—cytokines, chemokines, adhesion molecules, and acute-phase proteins—maintaining and exacerbating inflammation [42,45]. This proinflammatory profile likely promotes inflammation in placental vessels, which may be co-responsible for the development of PIH and preeclampsia [20,28]. Furthermore, hypoxia accompanies hypertension pathologies.

IL-17 increases the production of proinflammatory cytokines in the placenta [34,56,57]. IL-17 concentrations are significantly higher in women with preeclampsia than in healthy pregnant and non-pregnant women, especially in the early symptomatic form. At the same time, some studies do not confirm significant differences in serum between PE and controls [47]. A meta-analysis covering studies up to 2019 (IFN-γ, IL-1, IL-17, IL-22) also indicates a lack of consistent differences for circulating IL-17, and its authors recommend caution in interpretation due to the varying methods, type of biological material, population heterogeneity, and different analytical strategies [58]. Although we did not measure endothelin (ET) in our study, we consider it a potential marker and effector of vascular pathways in PIH: IL-17 may promote MMP activation and ET-1 maturation [59,60], and higher ET-1 levels and their association with symptom severity have been demonstrated in pregnant women with hypertension [55,59]. Endothelin (ET) itself is one of the most potent vasoconstrictor factors known [59]. Its secretion increases, among others, with endothelial damage, heat stress, and exposure to toxins [55]. IL-17 has also been shown to induce overexpression of metalloproteinases (MMPs), which convert inactive precursors (prepro-ET-1, proET-1) into biologically active endothelin-1 [59,60]. Consistent with this, higher ET-1 levels were found in amniotic fluid, fetal circulation, and serum of women with PIH compared to controls [59], and circulating ET-1 levels positively correlate with the severity of clinical symptoms [55]. Therefore, endothelin is considered a marker of endothelial damage in gestational hypertension and is also helpful in assessing the severity of preeclampsia.

In this context, abnormal IL-17F expression is associated with inflammatory and autoimmune diseases; its overproduction by Th17 cells can activate NK and cytotoxic lymphocytes, which, by attacking trophoblast, disrupt placental vascular remodeling and promote abnormal blood pressure regulation during pregnancy [61]. Logiodice et al. found no significant differences in IL-17F levels produced by peripheral blood lymphocytes and decidual lymphocytes in women with normal pregnancies [62]. In our study, the percentage of Th17 cells co-producing IL-21 and IL-22 was more than twice as high in patients with pregnancies complicated by hypertension as in healthy pregnant women; we also observed a weak positive correlation with maternal age. Although Th17 cells with intracellular IL-17F/IL-22 co-expression constituted <3% of the CD4^+^ population overall, the more than threefold increase compared to controls suggests their involvement in hypertension in pregnancy. Importantly, an increased percentage of Th17 cells secreting IL-22 in severe preeclampsia was previously described by Zhang et al. [28].

From a mechanistic perspective, IL-22, due to its immunoregulatory properties, acts through a heterodimeric receptor (IL-22R1/IL-10R2) and is involved in numerous inflammatory diseases [28,63]. IL-21, produced by Th17, has pleiotropic effects: it inhibits dendritic cell maturation, Th1 responses, and Treg functions, while enhancing Th17/Th2 pathways and stimulating the secretion of proinflammatory cytokines by macrophages [64]. It acts primarily through STAT3 activation; the absence of STAT3 abolishes the ability of IL-21 to initiate Th17 differentiation [65]. IL-21 also acts autocrinally on Th17 and regulates the functions of B lymphocytes, NK cells, and dendritic cells [66]; in vivo, it supports the humoral response, promoting the differentiation of B lymphocytes into plasma cells [65]. In vitro models indicate that IL-21 is produced by only a small percentage of human CD4^+^ cells (approximately 6.1% among IFN-γ^+^ cells and approximately 10% among IL-17^+^) [66], and Th17 selectively produce it, inducing the expression of RORγt, IL-17A, and IL-17F; this is also confirmed by microarray analyses (higher IL-21 production by Th17 vs. Th1/Th2) [65]. Clinical data are ambiguous: Poordast et al. (2017) found no differences in IL-21 concentrations between PE and healthy pregnant women but showed higher IL-21 levels in healthy non-pregnant women than in healthy pregnant women (*p* = 0.002) [67]; in URSA, IL-21 expression (RT-PCR) did not differ from healthy non-pregnant women [68]. In preeclampsia, a positive correlation was demonstrated between the percentage of Th22 and Th17 lymphocytes and the plasma IL-22 concentration and the percentage of Th22 cells; at the same time, CD4^+^IL-17A^+^IL-22^+^ cells were observed more frequently, although the plasma IL-22 level did not correlate with the number of circulating Th17 cells, suggesting that the source of IL-22 are both Th22 and Th17, with the share of Th17 in the plasma pool being small [28]. Another study confirmed the presence of IL-22 in most samples of maternal serum, umbilical cord blood and neonatal blood in normal pregnancies, with HCA and with PE. There were no statistically significant differences between the groups (*p* = 0.06); however, a trend towards higher IL-22 concentrations was observed in women with PE, as well as significantly higher IL-22 concentrations in cord blood and in neonates with PE compared to controls and HCA [69]. The authors interpreted this as a possible marker of placental dysfunction and initiation of repair processes at the maternal–fetal interface [69]. Biologically, IL-22 primarily exhibits antiapoptotic and pro-regenerative effects [69]; macrophages located in the decidua near apoptotic cells can remove dying cells and prevent the release of auto- and alloantigens [70]. Experimental data indicate that IL-22, via the IL-22R1/IL-10R2 receptor on the trophoblast, activates pathways promoting the expression of antiapoptotic and proliferative proteins, supporting trophoblast survival and maintaining pregnancy; reduced IL-22R1 expression in the villi may be associated with recurrent miscarriages [63]. The slight elevation of IL-22 observed in some patients with PE may reflect impaired trophoblast–decidua interaction and secondary overproduction of this cytokine [71]. Analysis of a panel of five cytokines (IL-17, IL-22, IL-21, IL-10, TGF-β) showed that plasma concentrations of most mediators were elevated in PE compared to healthy pregnant women in the second trimester, as well as in the third trimester (except TGF-β); at the same time, IL-21 and IL-22 were significantly higher in extracellular vesicles (EVs), indicating the involvement of EV signaling in the generalized inflammatory response in PE [20,72]. It is also known that impaired placental blood flow in PE increases apoptosis and EV release [57]. It is worth noting that there are also contradictory data—in one study, serum IL-22 concentrations were significantly lower in women with PE than in normal pregnancy (*p* < 0.001) [64]. Regardless of the discrepancies, the analysis of a broad panel of 53 mediators suggests that IL-22, CCL22, and the IL-2/IL-4 ratio may aid in the diagnosis of PE and even in differentiating preeclampsia from gestational hypertension.

### Limitations of the Study

Despite its important conclusions, our study has certain limitations. The first is that the experience and its analysis are a prospective, case–control observational design, which allows for the detection of associations, but has limitations in terms of causal inference. Another issue is blood sampling. Due to the patients’ presentation at weeks later than 37 of pregnancy, and despite strict adherence to inclusion and exclusion criteria, blood was collected at various weeks of pregnancy. It should be noted that we adhered to the dates specified in the patient inclusion criteria. This type of situation could have influenced potential differences between trimesters and potentially affected cellular composition and cytokine profiles. The third limitation was the differences in BMI/body weight between the study groups. Another issue is the long-term nature of the study, associated with the extended patient recruitment process. Despite consistent procedures, equipment, and antibody panels, this may pose the risk of subtle differences in the datasets obtained. A final point is that we analyzed peripheral blood, not tissues at the maternal–fetal interface, so conclusions about local placental immunology are more indirect.

## 5. Conclusions

The obtained results confirm the critical role of T helper cell responses in the pathogenesis of blood pressure disorders in pregnancy, including pregnancy-induced hypertension and preeclampsia. In women with pregnancies complicated by hypertension, a significantly higher percentage of activated Th17 lymphocytes was observed, along with a shift in the immune balance toward a proinflammatory response, as reflected by an abnormal Th17/Treg ratio. The accompanying dominance of proinflammatory cytokines may be a key mechanism contributing to the development of systemic inflammation and endothelial dysfunction, leading to the clinical symptoms of PIH and PE.

In light of the obtained data, the increased percentage of Th17 lymphocytes characterized by the simultaneous expression of IL-21 and IL-22 appears to be of particular importance, as it may constitute a potential immunological predictive marker for gestational hypertension. Further multicenter prospective studies are needed to determine the diagnostic usefulness and prognostic value of this approach in identifying women at high risk of developing this complication.

## Figures and Tables

**Table 1:** Characteristics and comparison of the study and control groups in terms of family history of hypertension as well as blood pressure measurement results.

Variable	Control Group (*n* = 48)	Study Group (*n* = 60)	*p*-Value
Family history			
No	41 (85.4%)	41 (85.4%)	41 (68.3%)
Yes	7 (14.6%)	19 (31.7%)	0.0662
Pre-pregnancy systolic blood pressure (SBP) [mmHg]	Median: 120 IQR: [110–120] Range: 90–140	Median: 120 IQR: [120–130] Range: 102–145	0.0014 *
Diastolic blood pressure before pregnancy (DBP) [mmHg]	Median: 71.5 IQR: [68–80] Range: 50–90	Median: 80 IQR: [80–85] Range: 60–115	<0.0001 *
Systolic blood pressure in pregnancy (SBP) [mmHg]	Median: 120 IQR: [113.5–122.5] Range: 90–140	Median: 155 IQR: [145–165] Range: 130–210	<0.0001 *
Diastolic blood pressure in pregnancy (DBP) [mmHg]	Median: 80 IQR: [70–80] Range: 60–90	Median: 95.5 IQR: [90–105] Range: 85–180	<0.0001 *

* Statistically significant values (*p* < 0.05); IQR—interquartile range.

**Table 2:** Comparison of immunological parameters of peripheral blood between the control group and the study group.

Parameter	Control Group (n = 48)	Study Group (n = 60)	*p*
Percentage of peripheral blood lymphocytes [%]	Median: 45.3 IQR: [37.0–60.9] Range: (14.6–72.9)	Median: 47.5 IQR: [39.4–54.7] Range: (16.6–79.1)	0.9501
Treg (CD4+CD25+FoxP3+) [%]	Median: 2.4 IQR: [0.9–3.9] Range: (0.2–19.0)	Median: 1.9 IQR: [0.8–3.4] Range: (0.2–8.1)	0.1677
Th17 (CD4+IL-17A+) [%]	Median: 1.8 IQR: [1.2–2.8] Range: (0.6–9.4)	Median: 6.8 IQR: [3.8–9.8] Range: (0.5–22.6)	<0.0001 *
Th17 (IL-17F+IL-21+) [%]	Median: 1.2 IQR: [0.7–2.1] Range: (0.1–8.1)	Median: 1.2 IQR: [0.6–3.1] Range: (0.3–7.5)	0.8368
Th17 (IL-17F+IL-22+) [%]	Median: 0.7 IQR: [0.3–2.1] Range: (0–8.2)	Median: 2.7 IQR: [1.6–5.7] Range: (0.1–43.1)	<0.0001 *
Th17 (IL-21+IL-22+) [%]	Median: 0.8 IQR: [0.2–1.5] Range: (0–10.3)	Median: 1.7 IQR: [0.8–5.8] Range: (0.1–28.8)	0.0001 *
Treg/Th17 ratio	Median: 19.3 IQR: [13.0–32.2] Range: (3.2–66.8)	Median: 5.5 IQR: [3.7–8.5] Range: (1.2–87.8)	<0.0001 *

* Statistically significant values (*p* < 0.05); Treg—regulatory T cells; Th17—type 17 helper lymphocytes; IQR—interquartile range.

**Table 3:** Correlations between the percentages of regulatory and helper T cells (Th17) expressing specific antigens and selected demographic and clinical variables.

Variable	Percentage of Peripheral Blood Lymphocytes		Percentage of Activated Th17 Lymphocytes (CD4+IL-17A+)		Percentage of Th17 Lymphocytes with Intracellular Expression of IL17F and IL21		Percentage of Th17 Lymphocytes with Intracellular Expression of IL17F and IL22		Percentage of Th17 Lymphocytes with Intracellular Expression of IL21 and IL22		Treg/Th17 Ratio	
	Rho	*p*	Rho	*p*	Rho	*p*	Rho	*p*	Rho	*p*	Rho	*p*
Mother’s age [years]	0.043	0.7485	−0.116	0.3922	−0.157	0.2434	0.193	0.1512	0.335	0.0109 *	0.108	0.4246
Number of pregnancies	−0.077	0.5617	−0.025	0.8560	−0.139	0.3016	0.077	0.5669	0.236	0.0774	−0.003	0.9846
Number of births	−0.057	0.6654	0.013	0.9220	−0.153	0.2551	0.020	0.8831	0.178	0.1858	−0.019	0.8910
Number of miscarriages in the history	−0.102	0.4458	−0.082	0.5457	−0.030	0.8256	0.136	0.3161	0.199	0.1414	0.055	0.6874
Week of pregnancy (hbd) on the day of sample collection	0.149	0.2591	−0.003	0.9823	0.263	0.0484 *	0.325	0.0136 *	0.124	0.3592	0.037	0.7840
Week of pregnancy (hbd) on the day of delivery	0.185	0.1612	−0.011	0.9331	0.234	0.0803	0.333	0.0114 *	0.133	0.3236	0.041	0.7627
Newborn body weight [g]	0.221	0.0926	−0.072	0.5962	0.082	0.5436	0.328	0.0128 *	0.157	0.2448	0.096	0.4791
Newborn’s body length [cm]	0.153	0.2514	0.022	0.8737	0.175	0.1969	0.404	0.0020 *	0.177	0.1931	−0.006	0.9671
APGAR in 1 min	0.310	0.0213 *	0.123	0.3807	−0.176	0.2062	0.226	0.1029	0.065	0.6441	−0.067	0.6358
APGAR in 3 min	0.331	0.0177 *	0.054	0.7105	−0.013	0.9283	0.265	0.0627	0.049	0.7376	−0.002	0.9894
APGAR in 5 min	0.410	0.0019 *	0.136	0.3333	0.047	0.7393	0.182	0.1932	0.022	0.8747	−0.037	0.7941
Mother’s weight before pregnancy [kg]	0.248	0.0588	−0.076	0.5723	−0.321	0.0150 *	−0.057	0.6722	−0.057	0.6756	0.164	0.2221
Current mother weight (at hbd of material collection) [kg]	0.175	0.1848	0.003	0.9802	−0.132	0.3272	−0.140	0.2979	−0.082	0.5459	0.065	0.6333
BMI before pregnancy [kg/m^2^]	0.207	0.1161	−0.077	0.5673	−0.174	0.1953	−0.092	0.4981	−0.139	0.3015	0.166	0.2167
BMI during pregnancy (at the time of material collection) [kg/m^2^]	0.140	0.2896	−0.027	0.8443	−0.062	0.6459	−0.165	0.2208	−0.146	0.2787	0.069	0.6103
SBP before pregnancy [mmHg]	−0.231	0.0809	0.134	0.3230	0.092	0.4989	0.235	0.0811	0.014	0.9167	−0.210	0.1206
DBP before pregnancy [mmHg]	−0.154	0.2488	0.100	0.4632	−0.021	0.8799	0.103	0.4498	−0.055	0.6897	−0.103	0.4481
SBP in pregnancy [mmHg]	−0.016	0.9056	−0.011	0.9328	−0.145	0.2818	−0.057	0.6750	0.015	0.9123	−0.131	0.3325
DBP in pregnancy [mmHg]	−0.175	0.1850	0.133	0.3256	−0.250	0.0604	0.025	0.8510	0.072	0.5962	−0.135	0.3163

hbd—week of gestation; Th17—type 17 helper T cells; Treg—regulatory T cells; CD4^+^IL-17A^+^—activated Th17 lymphocytes expressing IL-17A; IL-17F/IL-21/IL-22—cytokines assessed intracellularly by flow cytometry; “percentage of peripheral blood lymphocytes”—% of all lymphocytes in peripheral blood; “percentage of activated Th17 (CD4^+^IL-17A^+^)”—% of Th17 cells with IL-17A; “percentage of Th17 expressing IL-17F & IL-21/IL-17F & IL-22/IL-21 & IL-22”—% of Th17 cells co-expressing the indicated cytokines; Treg/Th17 ratio—ratio of Treg to Th17; Rho—Spearman’s rank correlation coefficient (−1 to +1); *p*—significance value (asterisk * indicates *p* < 0.05). Clinical variables: Mother’s age—mother’s age [years]; Number of pregnancies/births/miscarriages—number of pregnancies/births/miscarriages [n]; Week of pregnancy (hbd) on sampling/delivery—week of pregnancy on the day of collection/on the day of delivery [week]; Newborn body weight/length—weight [g]/length of the newborn [cm]; APGAR in 1/3/5 min—Apgar score in the 1st, 3rd, and 5th minute [0–10]; Mother’s weight before pregnancy/current—mother’s weight before/current pregnancy [kg]; BMI before/during pregnancy—body mass index [kg/m^2^]; SBP/DBP before pregnancy—systolic/diastolic blood pressure before pregnancy [mmHg]; SBP/DBP in pregnancy—systolic/diastolic blood pressure in pregnancy [mmHg].

## Data Availability

The data presented in this study are available on request from the corresponding author. The data are not publicly available due to privacy and ethical restrictions.

## Abbreviations

The following abbreviations are used in this manuscript:

L-17A	Interleukin-17A
IL-17F	Interleukin-17F
IL-21	Interleukin-21
IL-22	Interleukin-22
PBMC	Peripheral Blood Mononuclear Cells
PE	Preeclampsia
PIH	Pregnancy-Induced Hypertension
Th17	T helper 17 cells
Treg	Regulatory T cells
